# Mapping the GDF15 Arm of the Integrated Stress Response in Human Cells and Tissues

**DOI:** 10.1101/2025.01.31.635929

**Published:** 2025-02-01

**Authors:** Janell LM Smith, Kamaryn Tanner, Jack Devine, Anna S Monzel, Alan A Cohen, Martin Picard

**Affiliations:** 1 Department of Psychiatry, Division of Behavioral Medicine, Columbia University Irving Medical Center, New York, NY 10032, United States; 2 Robert N Butler Columbia Aging Center, Mailman School of Public Health, New York, NY 10032, United States; 3 Department of Neurology, H. Houston Merritt Center for Neurological and Mitochondrial Disorders, Columbia University Irving Medical Center, New York, New York 10032, United States; 4 Department of Environmental Health Sciences, Mailman School of Public Health, New York, NY 10032, United States; 5 New York State Psychiatric Institute, New York, NY 10032, United States

**Keywords:** ISR, GDF15, OxPhos, SURF1, energetic stress

## Abstract

Mitochondrial stress activates the integrated stress response (ISR) and triggers cell-cell communication through secretion of the metabokine growth differentiation factor 15 (GDF15). However, the gene network underlying the ISR remains poorly defined, particularly across metabolically diverse cellular states and tissues. Using RNAseq data from fibroblasts subjected to metabolic perturbations, we develop an *ISR*^*GDF15*^ *index* quantifying the GDF15 arm of the ISR activation in human cells. Validation of *ISR*^*GDF15*^ *index* across 44 postmortem human tissues illustrates how this index can be applied to investigate tissue-specific and age-related ISR activation.

## Main Text

Cells sense reductive stress via the integrated stress response (ISR). The ISR activates the gene *GDF15*, which encodes a secreted cytokine/metabokine elevated in several chronic diseases [1, 2], with aging [3], and particularly strongly in primary mitochondrial diseases caused by defects in the oxidative phosphorylation (OxPhos) system [4, 5]. However, the ISR is pleiotropic and triggers multiple cellular processes[6]. As a result, there is no consensus on the gene network that constitutes the ISR in human cells, or whether the same set of genes operate in metabolically diverse organs or tissues.

**Figure 1.**
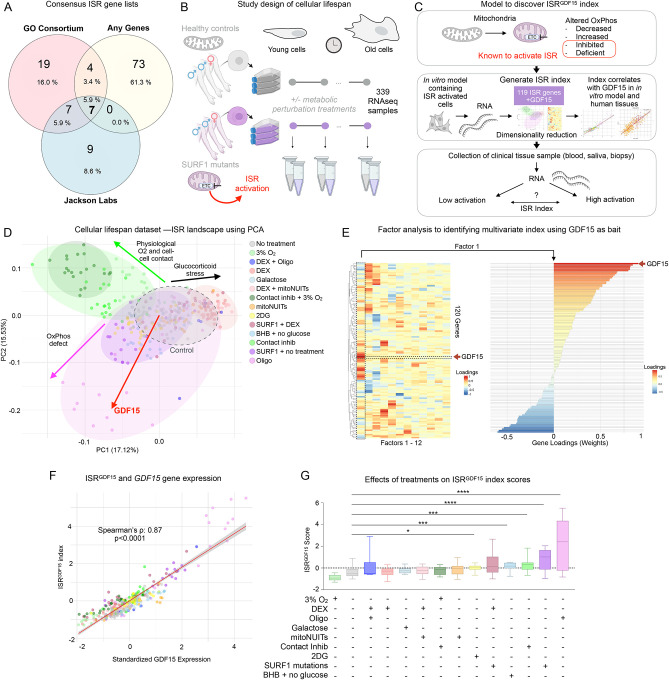
Generation of an ISR index using primary human fibroblasts. (A) Schematic of compiling ISR related genes, and showing minimal overlap between pathway lists. All 119 genes + *GDF15* will be used as a complete ISR list (B) Schematic of previously published work, where multiple healthy donors and mitochondrial disease patients donated fibroblasts, which were then cultured across their replicative lifespans up to 260 days. Cells were subjected to various treatments, including one that perturbed the ability of the cells to perform OxPhos, and samples were taken for bulk RNAseq, totaling 339 samples. (C) Model of generating ISR index. (D) Principal component analysis of the 339 samples using the119 genes from (A), plus *GDF15*. Red arrow denotes the loading vector of *GDF15* (E) Factor analysis containing *GDF15* in the gene list shows one factor strongly associated with *GDF15*, genes co-regulated with *GDF15* shown to the right. (F) Correlation of factor most co-regulated with *GDF15* (Factor 1) chosen to correlate each sample’s Factor 1 score vs its standardized *GDF15* expression. (G) Boxplots of ISR^GDF15^ scores for each condition, sorted by ascending median score subjected to Kruskal-Wallis test followed by a Dunn’s test for multiple comparisons. Oligo=Oligomycin, DEX= dexamethasone, mitoNUITs=mitochondrial nutrient uptake inhibitors, 2DG=2-Deoxy-D-glucose, BHB = β-hydroxybutyrate, contact inhib=contact inhibition.

To identify ISR-related genes linked to inter-cellular GDF15 signaling, we queried multiple ISR gene lists (total 119 genes) [7–9](Figure 1A), and examined their co-expression in a cellular lifespan system dataset [10]. Sensitivity analyses were performed on each gene list separately, as well as a randomly selected set of 119 genes, and demonstrated the superiority of utilizing the combined 119 genes from each list, plus *GDF15*, in the fibroblast dataset. This longitudinal transcriptomic dataset includes 339 fibroblast samples collected from either healthy or mitochondrial disease donors with mutations in the OxPhos complex IV assembly factor gene *SURF1* (Figure 1B). Each cell line was exposed to up to 11 metabolic perturbations including an inhibitor of the OxPhos system, oligomycin (Oligo), glucocorticoid agonist dexamethasone (DEX), mitochondrial nutrient uptake inhibitors (mitoNUITs), glucose deprivation plus β-hydroxybutyrate (BHB), low oxygen (3%), and contact inhibition [10]. OxPhos defects are potent triggers of the ISR in other datasets [11] and in this dataset [12], making this dataset and the variety of conditions an ideal testbed to examine gene expression signatures of the GDF15 arm of the ISR in human cells (Figure 1C).

To computationally isolate co-regulated ISR genes, we queried bulk gene expression RNAseq data using *GDF15* as a bait; we included *GDF15* alongside all 119 ISR genes from the three ISR gene lists, and performed dimensionality reduction analyses to explore the patterns of gene expression within our samples, focusing on the patterns most strongly associated with *GDF15*. Exploring the gene expression landscape across all treatments simultaneously using principal component analysis (PCA) (Figure 1D) identified a major axis of genes co-regulated with *GDF15* (principal component 2). Supplemental Figure S1A shows the loadings for each gene on each of the first two principal components. We next applied factor analysis to more quantitatively isolate the transcriptional signature of this *GDF15*-related ISR axis. This approach converged on an optimal solution containing 12 factors (i.e., sets of loading or “weights” applied to each of the 119 genes plus *GDF15* for a total of 120 genes) that collectively account for a maximal portion (73.1%) of the shared variance within this dataset (Figure 1E). The relation of principal components and each factor is quantified in Supplemental Figure S1B. One factor (Factor 1) – hereafter referred to as the *ISR*^*GDF15*^ *index* – explained 12.9% of the variance and was most strongly correlated with *GDF15* expression (Spearman’s rank correlation coefficient (ρ) = 0.87, p<1×10^−15^), establishing construct validity for the calculated *ISR*^*GDF15*^ *index* (Figure 1F).

To gain insight into the individual component genes of the *ISR*^*GDF15*^ *index*, we compared the genes most strongly associated (positively or negatively) with *GDF15*. The genes contributing most strongly to the *ISR*^*GDF15*^ *index* were enriched for positive regulation of endoplasmic reticulum unfolded protein response [top 10^positive^: *DDIT3*, *PPP1R15A*, *ERN1*, *GADD45A*, *CBX4*, *BBC3*, *SLC3A2*, *SIAH2*, *CEBPB*, *TRIB3*]. In contrast, negatively-weighted genes for the *ISR*^*GDF15*^ *index* were enriched for regulation of translation and translation initiation [top 10^negative^: *PPP1CA*, *APAF1*, *SLC35A4*, *FOS*, *IMPACT*, *NAIP*, *BIRC5*, *EIF2B1*, *NARS1*, *GCN2*] (see Supplemental Table 2 annotations and known functions of each gene). These results are consistent with the notion that cellular processes operate under energy constraints; while the activation of some energy-depending processes are upregulated, other processes must be downregulated[13, 14]. Therefore, cells activating the GDF15 signaling ISR arm may generally downregulate protein synthesis, as previously observed [15], despite known increases in specific proteins, such as in the case of GDF15 [12, 16].

Functionally, as expected relative to untreated control cells, the *ISR*^*GDF15*^ *index* was significantly induced in oligomycin-treated cells (large effect size, g = 2.13, p< 0.001) and in SURF1-mutant cells (g = 1.91, p<0.001, Figure 1G). The conditions of no glucose with β-hydroxybutyrate (g = 1.33, p<0.001), and the glycolysis inhibitor 2-deoxyglucose (g = 0.97, p<0.05) also increased the *ISR*^*GDF15*^ *index*, which could be anticipated due to glucose deprivation as a known ISR activator. Contact inhibition increasing the index (g = 1.23, p<0.001) was at first surprising; it was expected to be more physiologically similar to what cells experience *in vivo*, allowing for a more physiological skin cell culture. However, because the culture protocol in place only called for a media change once a week, matching the passaging of the non-contact inhibited cells, the higher density of cells throughout the week likely used the nutrients at a faster rate. Therefore, the contact inhibition group was likely being starved of nutrients, thus activating the ISR. To our further surprise, the low oxygen level (3%) treatment was not an activator of the ISR, as would be expected of a hypoxic treatment. However, upon literature investigation, what had been considered a hypoxic treatment for these fibroblast cells was, in fact, likely a physiological oxygenation level, for *in vivo* skin has oxygenation levels between 1.1–4.6% [17], and thus is likely to be a better control than our control group, which is cultured at 21% oxygen. Together, these data demonstrate the existence of a relatively small subset of inducible positively and negatively *GDF15*-related ISR genes induced by mitochondrial OxPhos perturbations.

**Figure 2.**
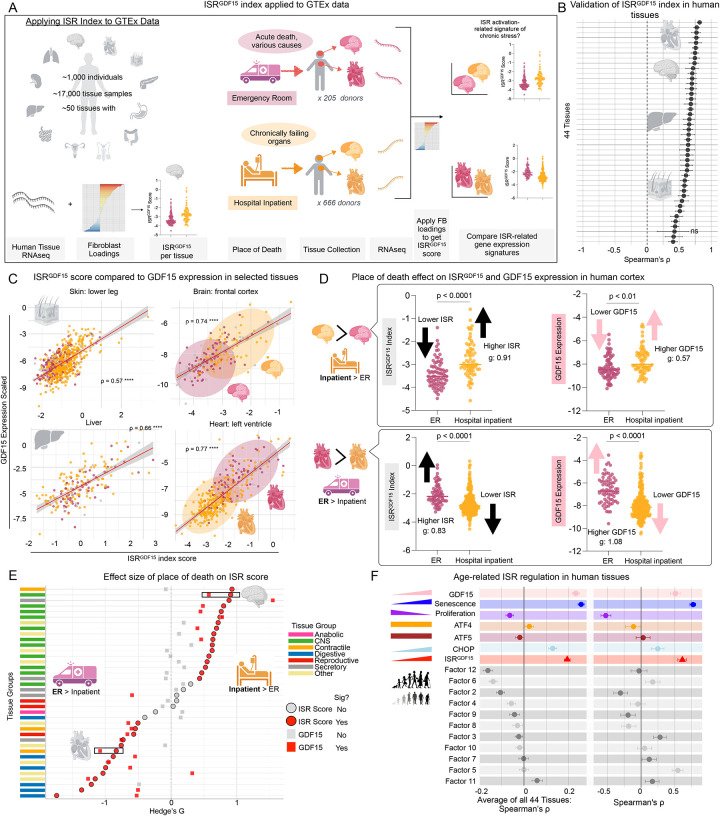
ISR index applied to human GTEx dataset. (A) Schematic of human GTEx dataset containing a variety of human organ samples, and applying the fibroblast generated index to the human transcriptomic data to get the human ISR^GDF15^ index scores for each tissue. (B) ISR^GDF15^ index correlations with *GDF15* across all the tissue samples expressing all of the 120 genes in the ISR index (Spearman’s ρ, +/− 95% confidence intervals). (C) Select tissue correlations. (D) ISR^GDF15^ or *GDF15* expression for each place of death, significance determined via Wilcoxon rank-sum test. (E) Effect size using Hedge’s G to compare each tissue’s ISR activation score for each place of death - hospital inpatient greater than ER on right, ER greater than hospital inpatient towards the left. Circles denote ISR score, squares *GDF15* expression, red as significant, gray non-significant. Wilcoxon rank-sum test was used to compare either the ISR score or *GDF15* expression in HI to ER, with BH method for multiple test correction. (F) Average Spearman’s correlations (+/− SEM) of all 44 tissues comparing the age of donor with the chosen gene(s) or ISR index score or factor scores (left) and fibroblasts’ age correlated with the chosen genes or ISR index score or factor score (+/− 95% confidence interval) (right). ISR^GDF15^ average tissue correlation denoted with a red triangle.

To pressure test this *ISR*^*GDF15*^ *index* outside the dish and examine whether it is conserved in the human body, we use the Genome-Tissue Expression (GTEx) project, which contains RNAseq samples of up to 17 different organs from around 1,000 donors [18](Figure 2A **left**). We first asked whether the *ISR*^*GDF15*^ *index* also was correlated with the bait *GDF15* gene when applied on human organs and tissues. This is expected to be the case if the internal correlation structure and loadings of the genes composing the *ISR*^*GDF15*^ *index* is similar across datasets. Focusing on tissues with a sample size >20, we observed a significant correlation between the *ISR*^*GDF15*^ *index* and *GDF15* in 43 out of 44 (97.7%) human tissues (ρ = 0.41–0.82, p<0.05, Figure 2B). This suggests that the genetic architecture of the *GDF15*-related arm of the ISR is at least partially conserved between fibroblasts *in vitro* and human tissues *in vivo*.

As an alternative test of validation, given the well-known upregulation of GDF15 with age, but not necessarily of the ISR per se, we sought to examine the sensitivity of the *ISR*^*GDF15*^ *index* to age. We examined the ISR-related gene expression as a function of age across all human tissues in GTEx (range 20–70, average 53 years), which showed that as expected *GDF15* is consistently more highly expressed in older individuals in 33 of 44 tissues (average ρ = 0.23, p<0.001, Figure 2F). This is lower than expected given GDF15’s established correlation with age [3]. The variability across the health status of all individuals as well as averaging across 44 tissues may be masking the correlation of *GDF15* with age. We also confirmed that senescence-associated genes (averaged tissue expressions of *CDKN1A*, *CDKN2A*, *CCND2*) were upregulated whereas replication-related genes (averaged tissue expressions of *TOP2A*, *RRM2*, *MKI67*) were downregulated with advancing age, further validating the cellular lifespan system [10] to probe age associations. Other single ISR genes *ATF4* and *ATF5* were unrelated to age; *CHOP* was mildly related to age (ρ=0.13) at about half the effect size as *GDF15*. The *ISR*^*GDF15*^ *index* was positively associated with age (ρ = 0.19, p<0.001), illustrating that the *GDF15* associated arm of the ISR is upregulated with age, and more strongly than individual genes classically referenced alone (or in conjunction with others) to denote activation of the ISR.

We then systematically examined the other 11 potential arms of the ISR isolated by factor analysis, ranked by negatively to positively age-related effect sizes across all GTEx tissues (Figure 2F). Four other ISR arms were significantly negatively associated with age (Factors 12, 6, 2, 4), and one (Factor 11) was upregulated with age. Applying the same analysis to aging human fibroblasts similarly showed both *GDF15* alone and the *ISR*^*GDF15*^ score were positively associated with age (ρ = 0.51 and 0.61 respectively), though the effect sizes were smaller than the senescence associated markers, as expected (ρ = 0.77). When we correlate the age-related effect sizes for each gene, ISR indices, and 11 factors between the *in vivo* and *in vitro* datasets, we find there is a moderate correlation (ρ = 0.69, p<0.01, Supplemental Figure S1C **left**), but an even stronger correlation (ρ = 0.93, p<0.01) when the 11 factors are not included (Supplemental Figure S1C **right**). This finding supports the notion that the *ISR*^*GDF15*^ signaling arm has an age-related association, due to the coherence between the two systems (*in vivo* and *in vitro*), similar to that of *GDF15* in both strength and direction. In other words, it supports that the *ISR*^*GDF15*^ *index* extracts most of the age-related signal of the ISR. The other factors may be important processes, but their age associations are weak.

In GTEx, some donors had died suddenly in the intensive care unit, while other individuals were hospital inpatients with more protracted death processes. To test whether ISR activation was higher in one group of donors compared to the other, we calculated the *ISR*^*GDF15*^ *index* for each tissue for each place of death: hospital inpatients (HI) and emergency room (ER). We focused on these two groups for two reasons: i) each group included a sufficient number of samples to allow robust comparisons of ISR activation; and ii) The ISR is activated in a time-dependent manner [6]. Thus, we hypothesized that longer hospital stays and pathology in HI would produce higher *ISR*^*GDF15*^ activation compared to ER donors, who on average would have less time to activate the ISR before death.

The expected ISR activation pattern was significantly observed only in a specific subset of tissues: brain tissues, skeletal muscle, skin, tibial nerve, and subcutaneous adipose tissue. On the other hand, donors from the ER had significantly higher *ISR*^*GDF15*^ score in a different subset of tissues: mammary breast, atrial appendage and left ventricle of the heart, prostate, thyroid, whole blood, aorta and coronary arteries, esophageal tissues, lung, transverse colon, and stomach (Figure 2E).

To investigate this discrepancy, we drew upon previous research by Mick et al, which demonstrated that the same stressor triggered differential ISR activation between myoblasts (actively proliferating) compared to myotubes (non-proliferative) [11]. To explore whether proliferation status could contribute to the differential ISR activation across human tissues, we calculated the proliferation score of each tissue [19] (see methods for details) and split the tissues into two groups: proliferative vs non-proliferative, using the median proliferation score to split the tissues. We then compared the ISR scores between the two groups based on proliferation, and saw a significant difference (g = 2.52, p < 0.001), with the proliferative tissues exhibiting higher ISR activation scores than the non-proliferative tissues (Supplemental Figure S1D). This was further validated by correlating ISR activation score with proliferation score of each tissue, resulting in a significant correlation (ρ = 0.78, p<0.001, Supplemental Figure S1E). Finally, when the groups are split by tissues showing a significantly higher ISR activation score for one group compared to the other (either HI to ER or ER to HI), we find the two groups have different proliferative indexes (g = 1.17, p<0.01, Supplemental Figure S1F). Ultimately, these analyses support the notion that proliferation status contributes to different ISR activation in a tissue dependent manner, and helps explain the distribution of tissues’ ISR activation according to place of death seen in Figure 2E.

Interestingly, the ISR index demonstrated higher sensitivity to sudden death vs chronic illness (or vice versa) compared to *GDF15* expression in tissues, reflected in 81.6% of tissues as significantly different ISR scores under these two scenarios compared to only 52.6% of tissues showing a significant difference of *GDF15* expression under the same scenarios (Chi square = 5.96, p = 0.015, Figure 2E). This is exemplified in the brain frontal cortex, in which the effect size of the ISR index score of sudden death to chronic illness is large (g = 0.91) while the *GDF15* effect size was only moderate (g = 0.57, Figure 2D), despite the strong correlation in this tissue between the *ISR*^*GDF15*^ *index* and *GDF15* expression (ρ = 0.74, Figure 2C). This supports the use of the *ISR*^*GDF15*^ *index* rather than *GDF15* alone as an indicator of ISR activation.

Together, this work i) challenges the monolithic understanding of the ISR in humans, showing instead that the ISR defined by popular gene lists [7–9] contains distinct sub-responses or sub-pathways. We also ii) identify a conserved set of *GDF15*-related canonical ISR genes upregulated by experimental mitochondrial perturbations. The identified *ISR*^*GDF15*^ *index* and *GDF15* itself are co-regulated across most human tissues. Finally, this work iii) quantitatively establishes other potential arms or components of the human ISR. In particular, our results (see Figure 2C–E) highlight potential tissue-specificity in ISR activation with disease and conditions leading up to death, calling for considerations of as of yet poorly defined tissue-specific regulatory mechanisms or downstream effects. Limitations of our approaches include their reliance on a linear combination of values (genes likely interact in non-linear ways [20], the use of post-mortem human tissues which could alter gene expression at least in the brain [21], the potential limited generalizability (despite the analyses across hundreds of *in vitro* conditions and timepoints and human tissues), and lack of validation of ISR activation on the protein level, which could be performed via western of phosphorylated eIF2alpha. Also, the genes in our consensus gene list are not an exhaustive list and could be missing other important genes (though these genes still perform robustly for the analyses generated). It is also possible that various tissues in the GTEx dataset have “contaminated” other tissues, especially adipose tissue which could affect specific gene expression values in some of the tissues more than others.

Overall, the general approach presented to produce multi-gene indices, and the *ISR*^*GDF15*^ *index* in particular provide a foundation for future research aiming to dissect the multifaceted biology of the human ISR in relation to health, disease and aging.

## Methods

### ISR Gene List Compilation

Gene lists for the ISR were gathered from three sources: GO Consortium, The Jackson Laboratory, and Any Genes by inputting “integrated stress response” in the search bars in their main websites [7–9]. The genes within their lists were merged together to make one master ISR gene list. Some genes had to be altered to fit the nomenclature of the specific transcriptomic dataset being used, as different gene aliases were used in each dataset for some genes, such as NARS1/NARS, WARS1/WARS, Igtp/IRGM. The genes found in each gene list are depicted in Supplemental Table 1. Information about the top 10 positively and negatively associated genes in the *ISR*^*GDF15*^ *index* was found by searching each gene in URL: https://www.genecards.org/.

### Fibroblast Lifespan Study

Lifespan study is explained in full detail in Sturm et al [10]. RNAseq data was downloaded from the shinyapp URL: https://columbia-picard.shinyapps.io/shinyapp-Lifespan_Study/. Fibroblast transcriptomic data was previously normalized via DEseq2, v1.30.1, and was log2 transformed. A total of 339 samples (excluding control line technical replicate data) was used for further analysis. Five healthy control lines and three patient-derived SURF1 mutant lines were cultured, with or without various metabolic treatments, as described in Sturm et al [10, 12].

### PCA

R version 4.4.1 with R studio was used to perform PCA (and all subsequent statistical analyses). The prcomp function was used to generate PCA, (stats package v4.4.1) using 339 fibroblast samples and the master list of ISR genes plus *GDF15*.

### Factor Analysis

The same fibroblast dataset, 339 samples and 120 genes, were used in the factor analysis. The factanal function (stats package) was used, with a varimax rotation. Scree method was used to determine the number of factors, as a predetermined number was not hypothesized as the number of causes and outputs of the ISR have not all been determined to date. Of the 119 ISR genes, the *ISR*^*GDF15*^ *index* included 77 positive and 42 negative gene loadings, reflecting ISR genes positively and negatively related to *GDF15* (see Supplemental Table 3 for individual gene loadings).

### GTEx

RNA sequencing data was downloaded as gene expression read counts from GTEx Analysis V8. RNA integrity number (RIN) values of less than 6 were filtered out of the data. Trimmed Mean of M-values (TMM) values calculated using DGE method (BiocGenerics package, version 0.50.0). Tissue samples of less than 20 were excluded from the analysis, leaving the kidney cortex with the minimum tissue samples of 86. Four tissues were excluded from applying the *ISR*^*GDF15*^ *index*: bladder, brain cerebellar hemisphere, brain cerebellum and adrenal gland due to missing one or more genes from the ISR list. 44 tissues in total were included in the analyses. Three genes required changing aliases to match the fibroblast genes: *DELE1* was changed to *KIAA0141*, *WARS1* was changed to *WARS*, and *NARS1* was changed to *NARS*.

Spearman ρ values were calculated comparing *GDF15* to *ISR*^*GDF15*^ for each of the 44 tissues with a Bonferroni correction. Spearman’s ρs were also calculated with age against a variety of genes and factor scores, such as *GDF15*, *ATF4*, *ATF5*, *CHOP*. Genes for senescence (*CDKN1A*, *CDKN2A*, *CCND2*) [22] or proliferation (*TOP2*, *RRM2*, *MKI67*) [19] were averaged within each tissue then correlated with age. Averages across the 44 tissues for each comparison were generated (+/− SEM). Wilcoxon signed-rank test was used to see if the averaged tissue correlations were significantly different than 0 (Figure 2F).

When comparing either the *ISR*^*GDF15*^ *index* score of ER vs HI or *GDF15* ER vs HI, tissues with less than 20 samples per group (ER or HI), were filtered out. Six tissues were filtered out: kidney cortex, pancreas, small intestines of the ileum, spleen, uterus, and vagina for having less than 20 samples in the ER group. Wilcoxon rank-sum test was used, with Benjamini-Hochberg (BH) multiple comparisons correction using 76 as the number of total tests. Tissues of Figure 2E were split into the following groups: liver into Anabolic; all brain tissues into central nervous system (CNS); skeletal muscle, heart atrial appendage and heart left ventricle into Contractile; colon tissues, esophageal tissues, and stomach into Digestive; testis, ovary, and prostate into Reproductive; salivary gland, adipose subcutaneous and visceral omentum, thyroid, and pituitary into Secretory; skin lower leg and suprapubic, artery tibial, breast mammary, whole blood, artery coronary, lung, and artery aorta into Other.

Proliferation scores of all significant tissues from Figure 2E were generated as noted in Devine et al [19]. To split the tissues into two groups, proliferative vs non-proliferative, the median score of 0.63 was used. Spearman’s correlation of the proliferation scores compared to the *ISR*^*GDF15*^ *index* was performed using these tissues. The significant tissues from Figure 2E were used to generate another two groups, *ISR*^*GDF15*^ scores higher in HI vs ER or ER vs HI, and their proliferation scores used to compare the groups. Wilcoxon rank sum test was used to determine if there was a significant difference between the groups.

### Statistics

All correlations performed used spearman rank correlation. When comparing group differences of two groups, Wilcoxon rank sum tests were used. When noted, Bonferroni multiple testing correction was used. Otherwise, we used BH, a less stringent form of multiple testing corrections, for more exploratory questions. Effect size was determined via Hedge’s g, an unbiased analog of Cohen’s d [23]. Hedge’s g was calculated via the R function cohen.d from the package effsize, with the Hedge’s g correction applied. When comparing multiple treatment groups simultaneously in Figure 1F, Prism version 10.2.3 (347) was used to perform a Kruskal-Wallis test followed by Dunn’s test for multiple comparisons. Wilcoxon signed-rank test was also done in prism.

## Supplementary Material

Supplement 1 **Supplemental Figure 1.** (A) Biplot of gene vectors of PCA components 1 and 2. (B) Correlation matrix of first 12 components of the PCA and the 12 factors of the factor analysis. (C) Spearman’s correlations of the average Spearman ρs of all tissues comparing donor age to chosen genes, ISR^GDF15^ score, or other 11 factor scores (left), the same as (left) without 11 factors. (D-F) Only the 31 tissues that were significant in Figure 2E are included for these analyses (D) ISR^GDF15^ score of each tissue split into two groups based on their proliferation score, using the median proliferation score to split the groups. (E) Spearman correlation of ISR score to proliferation score. (F) Proliferation scores of each tissue split into two groups based on whether the tissue had a higher hospital inpatient (HI) or emergency room (ER) score from Figure 2E.

Supplement 2

## Data Availability

GTEx v8 RNAseq data is available for download at https://gtexportal.org/home/downloads/adult-gtex/bulk_tissue_expression.Cellular Lifespan Study fibroblast dataset can be downloaded at https://columbia-picard.shinyapps.io/shinyapp-Lifespan_Study/. R code for the analyses will be made available at www.github.com/mitopsychobio.
