# Supplementary material for: Mapping the GDF15 Arm of the Integrated Stress Response in Human Cells and Tissues
Source: bioRxiv. 2025 Jul 1:2025.01.31.635929. Originally published 2025 Feb 1. Preprint. [Version 2] doi: 10.1101/2025.01.31.635929 (PMC11838536; doi:10.1101/2025.01.31.635929)
Supplement: 1 [file NIHPP2025.01.31.635929V2-supplement-1.pdf]

bioRxiv preprint doi: https://doi.org/10.1101/2025.01.31.635929; this version posted July 1, 2025. The copyright holder for this preprint (which was not certified by peer review) is the author/funder, who has granted bioRxiv a license to display the preprint in perpetuity. It is made available under aCC-BY 4.0 International license.

GO Consortium	Any Genes	Jackson Labs
ARIH1	ACTG1	Abca7
ATAD3A	AKT1	Agr2
ATF4	APAF1	Agr2
BATF	ASNS	Atf4
BATF2	ATF3	Bok
BATF3	ATF4	Dele1
CEBPA	ATF5	Eif2ak1
CEBPB	ATF6	Eif2ak3
CEBPD	ATG10	Eif2ak4
CEBPE	ATG12	Eif2s1
CEBPG	ATG16L1	Gcn1
CREB3	ATG3	Impact
CREBZF	ATG5	Igtp
DDIT3	ATG7	Map3k20
DELE1	BBC3	Nck1
EIF2AK1	BCL2	Nck2
EIF2AK3	BCL2L11	Nfe2l2
EIF2AK4	BECN1	Oma1
EIF2S1	BIRC2	Ptpn1
FOS	BIRC3	Ptpn2
FOSL1	BIRC5	Qrich1
GCN1	BIRC7	Rpap2
HERC5	BNIP3L	Tmed2
IMPACT	CBX4	Tmem33
JUN	CEBPB	
JUNB	CEBPG	
LIG4	DDB1	
MAF	DDB2	
MAFB	DDIT3	
MAP3K20	DDIT4	
NFE2	EGLN2	
NFE2L2	EGLN3	
NFE2L3	EIF2AK1	
OMA1	EIF2AK2	
QRICH1	EIF2AK3	
RPAP2	EIF2AK4	
TMED2	EIF2B1	
	EIF2S1	
	EIF2S3	
	ERCC1	
	ERCC2	
	ERCC3	
	ERN 1	
	ERO1A	

bioRxiv preprint doi: https://doi.org/10.1101/2025.01.31.635929; this version posted July 1, 2025. The copyright holder for this preprint (which was not certified by peer review) is the author/funder, who has granted bioRxiv a license to display the preprint in perpetuity. It is made available under aCC-BY 4.0 International license.

FOS
GABARAP
GABARAPL2
GADD45A
GCN1
HSPA5
IBTK
LMO4
MAP1LC3A
MTOR
NAIP
NARS
NBR1
NFE2L2
NFKB1
NUPR1
PIK3CA
PMAIP1
PPP1CA
PPP1R15A
PPP1R15B
RPL7
SESN2
SIAH1
SIAH2
SLC1A4
SLC35A4
SLC38A2
SLC3A2
SLC7A11
SLC7A5
SQSTM1
TFE3
TFEB
TNFRSF10B
TRIB3
TXNIP
WARS
XBP1
XIAP

bioRxiv preprint doi: https://doi.org/10.1101/2025.01.31.635929; this version posted July 1, 2025. The copyright holder for this preprint (which was not certified by peer review) is the author/funder, who has granted bioRxiv a license to display the preprint in perpetuity. It is made available under aCC-BY 4.0 International license.

	Factor1	Factor2	Factor3	Factor4	Factor5
QRICH1	-0.029	0.496	-0.167	0.04	0.042
SLC1A4	0.217	0.156	0.53	0.376	-0.097
ABCA7	0.097	-0.108	0.578	-0.112	0.27
ATAD3A	-0.117	0.692	0.268	0.043	-0.486
SLC35A4	-0.554	0.091	0.382	0.108	0.201
AGR2	0.126	0.003	-0.189	-0.108	-0.01
GCN1	-0.299	0.595	0.543	0.054	-0.226
SLC3A2	0.669	0.284	0.044	0.535	-0.04
DELE1	0.12	-0.079	-0.336	0.192	0.033
SLC7A11	0.045	0.175	0.532	0.684	0.088
ATF4	0.437	0.153	-0.279	0.677	-0.194
HERC5	0.425	-0.321	-0.019	0.11	-0.201
SLC7A5	0.224	0.297	0.313	0.475	0.185
ARIH1	0.321	-0.013	0.331	0.138	0.328
NARS1	-0.39	0.142	0.552	0.521	0.279
RPAP2	-0.285	-0.796	0.142	-0.035	-0.131
APAF1	-0.589	0.03	0.067	-0.094	-0.325
BATF2	-0.156	-0.061	-0.308	0.121	0.152
BBC3	0.678	-0.255	-0.327	0.158	0.17
EIF2S1	-0.09	0.813	0.309	0.13	0.048
BCL2	0.519	-0.134	-0.515	0.03	-0.117
BATF3	-0.121	0.132	0.029	0.073	-0.501
BIRC2	-0.166	0.28	0.168	0.061	0.311
BOK	-0.042	-0.028	-0.283	0.038	-0.019
OMA1	0.07	0.001	0.121	-0.009	0.128
BIRC3	0.237	0.051	0.036	-0.025	-0.035
EIF2AK1	-0.098	0.51	0.011	0.113	0.272
BIRC5	-0.402	0.587	0.185	-0.071	-0.549
IMPACT	-0.474	-0.024	0.069	0.426	0.292
ATG3	0.392	0.779	0.049	0.102	-0.099
NFE2	-0.01	0	0.062	-0.003	-0.052
ATG5	-0.026	0.528	0.29	0.303	0.207
NFE2L3	0.171	0.07	-0.098	0.005	-0.574
ATG7	-0.38	0.462	-0.011	0.16	-0.21
CREBZF	0.059	-0.323	-0.316	-0.154	-0.168
GABARAP	0.405	0.025	-0.684	-0.058	0.342
EIF2AK4	-0.39	0.548	0.464	0.041	0.132
GABARAPL2	0.081	-0.015	-0.007	-0.084	0.887
CEBPE	0.089	-0.114	-0.037	-0.204	-0.215
NBR1	0.335	0.202	-0.069	-0.147	0.635
EIF2AK3	0.122	-0.436	-0.131	0.019	0.188
FOSL1	-0.18	0.355	0.079	-0.083	-0.388
SQSTM1	0.368	0.02	-0.351	0.071	0.213
FOS	-0.534	-0.453	-0.043	0.056	0.014
ASNS	0.086	0.278	0.329	0.728	0.125

bioRxiv preprint doi: https://doi.org/10.1101/2025.01.31.635929; this version posted July 1, 2025. The copyright holder for this preprint (which was not certified by peer review) is the author/funder, who has granted bioRxiv a license to display the preprint in perpetuity. It is made available under aCC-BY 4.0 International license.

CREB3	0.196	0.345	0.727	-0.025	-0.016
BECN1	0.054	0.583	0.415	-0.025	0.383
NFE2L2	0.594	0.071	-0.146	0.563	0.169
EIF2AK2	0.029	0.054	-0.208	0.183	0.154
JUN	-0.025	-0.582	-0.248	-0.184	0.312
MAP3K20	0.106	-0.088	-0.162	-0.024	-0.119
BATF	-0.012	-0.005	0.307	-0.007	0.005
MAF	0.066	-0.331	-0.75	-0.046	0.078
CEBPD	-0.118	-0.045	-0.053	0	0.137
MAP1LC3A	0.219	0.327	0.252	-0.062	0.469
LIG4	-0.338	-0.187	0.56	0.235	0.461
SLC38A2	-0.187	0.099	0.518	0.031	-0.08
DDB1	-0.17	0.446	0.117	0.028	0.23
IRGM	0.17	-0.485	-0.139	-0.091	-0.026
CEBPG	0.231	-0.082	-0.08	0.881	-0.142
DDB2	0.419	-0.142	0.136	0.018	0.477
CEBPB	0.634	0.197	-0.463	-0.165	0.08
DDIT4	-0.06	-0.115	0.357	0.144	0.052
JUNB	-0.277	-0.437	-0.286	-0.066	0.061
ATF6	0.016	0.165	0.367	0.367	0.461
TMED2	0.01	0.505	-0.062	0.432	0.107
HSPA5	0.488	0.13	-0.106	0.419	-0.414
CEBPA	0.09	-0.162	-0.067	-0.179	-0.028
NCK1	0.451	0.252	-0.329	0.186	0.226
DDIT3	0.844	-0.078	-0.303	0.042	-0.019
PIK3CA	-0.375	-0.046	0.172	0.163	0.589
MAFB	0.111	-0.323	-0.545	-0.219	-0.079
AKT1	-0.127	0.655	0.42	-0.116	-0.092
IBTK	-0.027	0.398	0.097	0.594	-0.055
NCK2	0.056	-0.103	-0.422	0.096	-0.019
MTOR	-0.112	0.503	0.624	0.269	0.103
ACTG1	0.159	0.654	-0.026	-0.046	-0.204
EIF2B1	-0.391	0.515	0.334	0.027	0.125
EIF2S3	0.069	0.516	-0.12	0.253	0.067
PPP1CA	-0.602	0.628	0.202	0.125	-0.272
RPL7	0.166	0.395	-0.61	0.226	0.209
PTPN1	-0.239	0.353	0.808	-0.048	0.19
SESN2	0.131	-0.259	0.259	0.771	0.03
WARS1	-0.126	0.121	0.018	0.896	0.1
BCL2L11	0.399	-0.36	-0.618	0.103	-0.025
PTPN2	0.199	0.449	0.014	-0.043	-0.331
ERN1	0.815	-0.313	-0.034	0.175	-0.003
ERO1A	-0.058	-0.183	-0.559	-0.247	0.101
PPP1R15A	0.819	0.114	-0.389	-0.087	-0.065
PPP1R15B	0.147	-0.132	0.108	0.33	-0.198
TRIB3	0.623	-0.073	-0.172	0.696	-0.045

bioRxiv preprint doi: https://doi.org/10.1101/2025.01.31.635929; this version posted July 1, 2025. The copyright holder for this preprint (which was not certified by peer review) is the author/funder, who has granted bioRxiv a license to display the preprint in perpetuity. It is made available under aCC-BY 4.0 International license.

XBP1	0.485	0.158	-0.145	0.706	-0.283
TMEM33	0.412	0.579	0.249	0.227	-0.022
ERCC1	0.083	0.331	-0.317	-0.043	-0.175
ERCC2	0.312	0.634	-0.047	0.006	-0.162
ERCC3	0.097	0.265	-0.114	0.161	0.277
GADD45A	0.814	0.247	0.184	0.058	0.045
BNIP3L	0.154	-0.173	-0.651	-0.233	0.424
ATF3	0.351	-0.133	-0.061	-0.001	-0.155
ATF5	0.195	-0.053	0.035	0.058	-0.202
CBX4	0.797	0.217	0.144	0.156	-0.032
EGLN2	0.277	0.283	-0.277	-0.115	-0.067
EGLN3	-0.026	-0.528	-0.323	-0.34	-0.01
LMO4	0.228	-0.063	-0.375	0.375	-0.008
NFKB1	-0.31	0.153	-0.352	0.006	-0.054
TFE3	0.439	0.67	0.01	0.037	0.013
TFEB	0.562	0.081	0.027	-0.226	0.032
TXNIP	-0.249	-0.013	0.192	0.185	0.423
BIRC7	0.204	-0.251	-0.103	-0.177	-0.234
NAIP	-0.461	-0.715	-0.183	0.16	-0.132
NUPR1	0.518	0.15	-0.003	0.254	0.438
PMAIP1	0.08	0.087	0.092	0.356	-0.47
SIAH1	0.45	-0.093	-0.71	0.203	-0.147
SIAH2	0.651	-0.042	-0.224	0.354	-0.037
TNFRSF10B	0.598	0.058	0.259	0.424	0.28
XIAP	0.56	0.015	-0.014	0.076	0.066
ATG10	0.022	-0.509	0.1	0.122	-0.084
ATG12	0.196	0.198	-0.074	0.053	0.664
ATG16L1	0.2	0.123	0.592	0.098	0.023
GDF15	0.902	0.057	0.026	0.231	0.12

bioRxiv preprint doi: https://doi.org/10.1101/2025.01.31.635929; this version posted July 1, 2025. The copyright holder for this preprint (which was not certified by peer review) is the author/funder, who has granted bioRxiv a license to display the preprint in perpetuity. It is made available under aCC-BY 4.0 International license.

Factor6	Factor7	Factor8	Factor9	Factor10	Factor11
0.386	-0.228	-0.058	0.39	0.161	-0.056
-0.103	0.286	-0.153	-0.123	0.327	0.132
0.041	0.31	-0.009	0.188	-0.089	-0.036
-0.028	-0.008	0.006	-0.088	-0.178	0.088
-0.189	0.092	-0.222	0.205	0.028	0.413
0.057	-0.072	-0.03	-0.06	-0.043	0.084
-0.063	0.066	0.005	0.047	-0.071	0.014
0.206	-0.17	-0.178	-0.077	0.01	0.023
-0.134	0.392	0.094	-0.248	0.145	0.258
0.174	-0.176	0.019	-0.024	0.174	-0.004
-0.045	-0.089	0.243	-0.215	-0.001	-0.078
0.446	-0.048	-0.041	0.196	0.1	0.003
0.27	-0.443	-0.041	-0.216	-0.051	-0.052
0.445	-0.044	0.208	0.09	0.319	-0.082
0.198	0.097	0.078	0.003	-0.072	0.119
-0.022	0.052	0.023	0.07	0.032	-0.141
-0.239	-0.327	0.139	-0.14	0.207	-0.264
0.413	-0.017	-0.216	0.327	-0.281	-0.005
0.066	-0.121	0.132	0.06	-0.298	-0.118
0.098	-0.051	-0.125	0.043	0.199	-0.132
0.155	-0.038	-0.047	0.148	-0.21	0.141
0.097	0.045	0.162	-0.025	-0.047	-0.15
0.386	-0.1	-0.051	-0.105	0.482	0.06
-0.208	-0.596	0.177	-0.444	-0.112	-0.008
0.018	0.746	-0.147	0.062	0.144	0.078
0.015	0.138	-0.447	0.004	0.215	0.176
0.374	0.384	-0.059	-0.081	0.344	0.113
-0.123	0.003	-0.235	-0.007	0.035	-0.006
0.137	-0.009	0.476	-0.164	0.006	-0.016
0.234	0.03	-0.143	0.072	-0.083	-0.046
0.052	0.052	-0.026	0.078	-0.035	-0.013
0.124	-0.085	-0.082	0.111	0.177	-0.102
-0.043	-0.057	0.185	0.057	0.127	-0.3
-0.076	0.01	-0.003	-0.008	-0.325	-0.201
-0.466	-0.014	0.231	-0.012	0.037	-0.23
0.392	0.046	0.075	-0.053	-0.142	0.058
0.018	-0.249	0.191	-0.1	-0.064	-0.135
0.2	0.033	-0.012	-0.066	-0.014	-0.029
0.168	0.009	-0.041	0.008	-0.049	0.017
-0.135	0.04	0.188	0.068	-0.03	-0.459
0.272	0.282	-0.07	0.182	0.155	0.483
0.507	-0.076	0.127	-0.031	-0.01	0.263
0.642	0.161	0.059	0.045	-0.411	0.086
-0.279	0.104	0.298	0.304	-0.053	0.324
-0.34	-0.199	-0.063	-0.034	0.088	-0.173

bioRxiv preprint doi: https://doi.org/10.1101/2025.01.31.635929; this version posted July 1, 2025. The copyright holder for this preprint (which was not certified by peer review) is the author/funder, who has granted bioRxiv a license to display the preprint in perpetuity. It is made available under aCC-BY 4.0 International license.

-0.147	-0.149	0.11	-0.181	-0.056	-0.103
0.182	-0.086	0.055	-0.074	-0.182	-0.31
0.187	0.036	0.05	0.127	0.218	0.013
0.619	0.029	-0.034	0.117	0.095	-0.008
-0.269	0.094	0.132	0.149	-0.165	0.171
0.044	-0.179	0.608	-0.086	0.173	-0.076
0.06	0.028	-0.184	0.012	-0.009	0.048
0.112	-0.122	-0.084	0.249	-0.305	0.029
-0.867	0.013	-0.039	0.158	-0.018	-0.017
-0.257	-0.098	0.086	-0.331	-0.095	-0.255
0.121	0.03	-0.048	0.125	0.189	0.152
-0.365	0.221	0.089	-0.02	0.511	-0.057
0.583	0.225	0.358	-0.122	-0.121	-0.057
0.095	-0.184	-0.034	-0.003	-0.013	-0.039
-0.147	0.021	0.001	0.017	0.057	0.021
0.085	-0.145	-0.024	-0.126	-0.014	0.007
-0.052	0.054	0.099	0.217	-0.348	-0.005
-0.683	0.065	0.024	-0.093	0.025	0.054
-0.224	0.104	0.276	0.355	-0.068	0.444
0.032	0.031	-0.118	0.003	0.278	0.474
0.396	0.141	-0.29	-0.09	0.357	0.155
0.311	0.095	-0.057	-0.185	0.083	0.266
-0.021	0.08	0.051	0.623	-0.063	0.018
0.152	0.128	-0.286	0.271	0.122	0.09
-0.025	0.114	-0.021	-0.089	-0.141	-0.036
0.039	0.117	0.007	-0.035	0.357	0.129
-0.095	0.105	-0.026	0.378	-0.039	0.336
-0.358	-0.184	-0.103	-0.052	0.076	0.046
0.194	0.048	0.188	-0.25	0.236	0.026
0.054	-0.25	0.108	-0.068	-0.079	0.187
0.107	0.019	0.122	-0.008	-0.037	0.144
-0.175	-0.083	0.347	-0.469	-0.072	-0.059
-0.31	-0.102	-0.044	-0.059	0.125	-0.023
0.034	0.643	-0.107	-0.079	-0.02	-0.009
0.109	-0.039	-0.069	0.027	-0.127	0.053
-0.155	0.04	0.202	-0.103	-0.015	-0.17
-0.023	-0.041	0.004	0.002	0.155	0.026
0.136	0.084	0.134	0.084	-0.168	0.027
0.153	0.084	-0.173	0.033	-0.119	0.023
0.071	-0.022	0.063	0.332	-0.168	-0.067
-0.26	0.205	-0.046	0.051	0.107	-0.052
0.05	0.185	0.025	-0.058	0.05	-0.064
0.24	0.046	-0.156	-0.251	0.17	0.396
-0.064	-0.081	0.067	0.137	-0.123	-0.042
-0.333	-0.087	0.49	0.461	0.041	0.074
0.012	0.111	-0.008	-0.066	-0.12	0.108

bioRxiv preprint doi: https://doi.org/10.1101/2025.01.31.635929; this version posted July 1, 2025. The copyright holder for this preprint (which was not certified by peer review) is the author/funder, who has granted bioRxiv a license to display the preprint in perpetuity. It is made available under aCC-BY 4.0 International license.

-0.036	0.058	-0.073	0.032	0.139	0.107
0.292	0.158	-0.183	0.071	0.15	0.139
-0.471	0.051	-0.069	-0.002	-0.077	0.05
0.003	-0.033	0.233	-0.47	-0.159	-0.014
0.189	-0.484	0.065	-0.048	0.129	0.09
0.092	0.11	-0.006	-0.073	0.113	0.046
-0.005	-0.067	0.127	-0.021	0.047	0.081
0.156	0.084	0.575	0.036	-0.005	0.17
0.077	0.651	0.207	-0.034	-0.045	0.047
-0.169	-0.045	0.293	-0.095	0.046	0.035
0.001	-0.198	-0.037	0.073	-0.606	-0.084
-0.235	0.004	-0.161	-0.044	0.074	0.037
0.024	0.142	0.315	-0.104	-0.035	-0.103
0.029	0.076	-0.231	0.578	-0.316	-0.011
0.03	-0.05	0.061	-0.089	-0.049	-0.227
-0.398	-0.111	-0.105	0.142	-0.039	-0.281
0.002	-0.075	-0.357	0.23	0.092	-0.026
-0.066	0.082	-0.153	-0.068	-0.081	0.103
0.015	0.085	0.099	0.039	0.139	0.057
-0.239	0.001	-0.086	-0.067	-0.366	0.081
0.205	-0.165	0.129	0.024	0.116	-0.029
0.118	-0.104	0.145	0.134	0.012	-0.112
-0.361	-0.086	0.141	-0.1	0.195	-0.02
0.363	-0.037	0.116	-0.104	0.021	0.03
0.518	0.203	0.116	-0.046	0.058	-0.063
-0.086	-0.356	0.172	-0.037	-0.014	-0.028
0.413	-0.091	-0.132	0.077	0.071	-0.128
-0.054	-0.101	0.009	-0.085	0.033	0.031
0.128	0.082	0.047	-0.042	-0.094	-0.019

bioRxiv preprint doi: https://doi.org/10.1101/2025.01.31.635929; this version posted July 1, 2025. The copyright holder for this preprint (which was not certified by peer review) is the author/funder, who has granted bioRxiv a license to display the preprint in perpetuity. It is made available under aCC-BY 4.0 International license.

Factor12
0.062
-0.013
-0.098
-0.061
-0.008
0.082
0.024
-0.063
-0.135
-0.193
-0.086
-0.127
0.067
0.107
0.024
0.135
0.155
-0.263
-0.193
0.144
-0.209
0.19
0.094
0.183
-0.118
-0.227
0.128
-0.045
-0.15
0.056
-0.281
0.005
-0.165
-0.296
-0.062
0.035
-0.18
0.164
0.093
-0.033
0.108
-0.227
0.032
0.003
0.025

bioRxiv preprint doi: https://doi.org/10.1101/2025.01.31.635929; this version posted July 1, 2025. The copyright holder for this preprint (which was not certified by peer review) is the author/funder, who has granted bioRxiv a license to display the preprint in perpetuity. It is made available under aCC-BY 4.0 International license.

-0.006
-0.04
0.012
-0.128
-0.005
-0.111
0.004
0.136
0.07
0.355
0.091
-0.003
0.006
0.019
-0.057
-0.432
0.047
0.128
0.053
-0.119
0.085
-0.085
-0.14
-0.193
0.051
0.143
-0.055
-0.064
0.077
0.215
-0.085
0.233
-0.024
0.018
0.012
-0.203
0.064
-0.088
0.08
0.075
-0.312
0.203
0.17
0.014
0.071
0.112

bioRxiv preprint doi: https://doi.org/10.1101/2025.01.31.635929; this version posted July 1, 2025. The copyright holder for this preprint (which was not certified by peer review) is the author/funder, who has granted bioRxiv a license to display the preprint in perpetuity. It is made available under aCC-BY 4.0 International license.

0.129
0.018
-0.228
0.202
-0.001
-0.218
0.179
0.086
0.274
-0.012
0.039
0.284
0.346
0.006
0.027
0.08
-0.146
0.182
-0.012
0.098
-0.483
0.146
0.249
-0.204
0.339
0.032
-0.005
0.04
-0.014

bioRxiv preprint doi: https://doi.org/10.1101/2025.01.31.635929; this version posted July 1, 2025. The copyright holder for this preprint (which was not certified by peer review) is the author/funder, who has granted bioRxiv a license to display the preprint in perpetuity. It is made available under aCC-BY 4.0 International license.

Top 10 positive genes

DDIT3
PPP1R15A
ERN 1
GADD45A
CBX4
BBC3
SLC3A2
SIAH2
CEBPB
TRIB3

bioRxiv preprint doi: https://doi.org/10.1101/2025.01.31.635929; this version posted July 1, 2025. The copyright holder for this preprint (which was not certified by peer review) is the author/funder, who has granted bioRxiv a license to display the preprint in perpetuity. It is made available under aCC-BY 4.0 International license.

Known functions of positive genes

Activated by ER stress, plays regulatory role in inflammatory response
Involved in autophagy, role in reversing global protein reduction initiated by ISR
Protein which senses unfolded proteins in the ER
Mediates activation of the p38/JNK pathway via MTK1/MEKK4 kinase
Plays a role in negative regulation of transcription by RNA polmerase II
Pro-apoptotic role
Assists biogenesis and trafficking of functional transporters to the plasma membrane
An E3 kinase involved in uibquitination of proteins, implicated in regulating cellular response to h
Regulates the expression of genes relating to immune and inflammatory responses, and other p
Inhibits activity of DDIT3, and acts as a negative feedback regulator of the ISR

bioRxiv preprint doi: https://doi.org/10.1101/2025.01.31.635929; this version posted July 1, 2025. The copyright holder for this preprint (which was not certified by peer review) is the author/funder, who has granted bioRxiv a license to display the preprint in perpetuity. It is made available under aCC-BY 4.0 International license.

Top 10 negative genes	Known functions of negative genes
PPP1CA	Involved in the regulation of many cellular processes, such
APAF1	A cytoplasmic protein that initiates apoptosis
SLC35A4	Plays a role in positive regulation of cellular respiration an
FOS	Associated with apoptosis, implicated in regulation of cell
IMPACT	Regulator of translation, promotes high levels of translatio
NAIP	Inhibits activities of caspase 3, 7, and 9, functions to preve
BIRC5	Promotes proliferation and inhibits apoptosis
EIF2B1	Component of the translation initiation factor 2B complex,
NARS1	Catalyzes attachment of asparagine to tRNA, essential rol
GCN2	Phosphorylates eIF2, resulting in down regulation of prote

bioRxiv preprint doi: https://doi.org/10.1101/2025.01.31.635929; this version posted July 1, 2025. The copyright holder for this preprint (which was not certified by peer review) is the author/funder, who has granted bioRxiv a license to display the preprint in perpetuity. It is made available under aCC-BY 4.0 International license.

ı as protein synthesis and cell division

d positive regulation of translation in response to stress
proliferation, differentiation, and transformation
n in response to a variety of stressors
ent apoptosis

regulator of protein synthesis
le in protein synthesis
in synthesis in response to low amino acid availability

bioRxiv preprint doi: https://doi.org/10.1101/2025.01.31.635929; this version posted July 1, 2025. The copyright holder for this preprint (which was not certified by peer review) is the author/funder, who has granted bioRxiv a license to display the preprint in perpetuity. It is made available under aCC-BY 4.0 International license.

Genes	Positive Loadings	Genes	Negative Loadings
GDF15	0.902	NFE2	-0.01
DDIT3	0.844	BATF	-0.012
PPP1R15A	0.819	JUN	-0.025
ERN 1	0.815	ATG5	-0.026
GADD45A	0.814	EGLN3	-0.026
CBX4	0.797	IBTK	-0.027
BBC3	0.678	QRICH1	-0.029
SLC3A2	0.669	BOK	-0.042
SIAH2	0.651	ERO1A	-0.058
CEBPB	0.634	DDIT4	-0.06
TRIB3	0.623	EIF2S1	-0.09
TNFRSF10B	0.598	EIF2AK1	-0.098
NFE2L2	0.594	MTOR	-0.112
TFEB	0.562	ATAD3A	-0.117
XIAP	0.56	CEBPD	-0.118
BCL2	0.519	BATF3	-0.121
NUPR1	0.518	WARS1	-0.126
HSPA5	0.488	AKT1	-0.127
XBP1	0.485	BATF2	-0.156
NCK1	0.451	BIRC2	-0.166
SIAH1	0.45	DDB1	-0.17
TFE3	0.439	FOSL1	-0.18
ATF4	0.437	SLC38A2	-0.187
HERC5	0.425	PTPN1	-0.239
DDB2	0.419	TXNIP	-0.249
TMEM33	0.412	JUNB	-0.277
GABARAP	0.405	RPAP2	-0.285
BCL2L11	0.399	GCN1	-0.299
ATG3	0.392	NFKB1	-0.31
SQSTM1	0.368	LIG4	-0.338
ATF3	0.351	PIK3CA	-0.375
NBR1	0.335	ATG7	-0.38
ARIH1	0.321	NARS1	-0.39
ERCC2	0.312	EIF2AK4	-0.39
EGLN2	0.277	EIF2B1	-0.391
BIRC3	0.237	BIRC5	-0.402
CEBPG	0.231	NAIP	-0.461
LMO4	0.228	IMPACT	-0.474
SLC7A5	0.224	FOS	-0.534
MAP1LC3A	0.219	SLC35A4	-0.554
SLC1A4	0.217	APAF1	-0.589
BIRC7	0.204	PPP1CA	-0.602
ATG16L1	0.2		

bioRxiv preprint doi: https://doi.org/10.1101/2025.01.31.635929; this version posted July 1, 2025. The copyright holder for this preprint (which was not certified by peer review) is the author/funder, who has granted bioRxiv a license to display the preprint in perpetuity. It is made available under aCC-BY 4.0 International license.

PTPN2	0.199
CREB3	0.196
ATG12	0.196
ATF5	0.195
NFE2L3	0.171
IRGM	0.17
RPL7	0.166
ACTG1	0.159
BNIP3L	0.154
PPP1R15B	0.147
SESN2	0.131
AGR2	0.126
EIF2AK3	0.122
DELE1	0.12
MAFB	0.111
MAP3K20	0.106
ABCA7	0.097
ERCC3	0.097
CEBPA	0.09
CEBPE	0.089
ASNS	0.086
ERCC1	0.083
GABARAPL2	0.081
PMAIP1	0.08
OMA1	0.07
EIF2S3	0.069
MAF	0.066
CREBZF	0.059
NCK2	0.056
BECN1	0.054
SLC7A11	0.045
EIF2AK2	0.029
ATG10	0.022
ATF6	0.016
TMED2	0.01
