# Supplementary material for: Mapping the GDF15 Arm of the Integrated Stress Response in Human Cells and Tissues
Source: bioRxiv. 2025 Feb 1:2025.01.31.635929. Preprint. [Version 1] doi: 10.1101/2025.01.31.635929 (PMC11838536; doi:10.1101/2025.01.31.635929)

Supplemental Figure 1

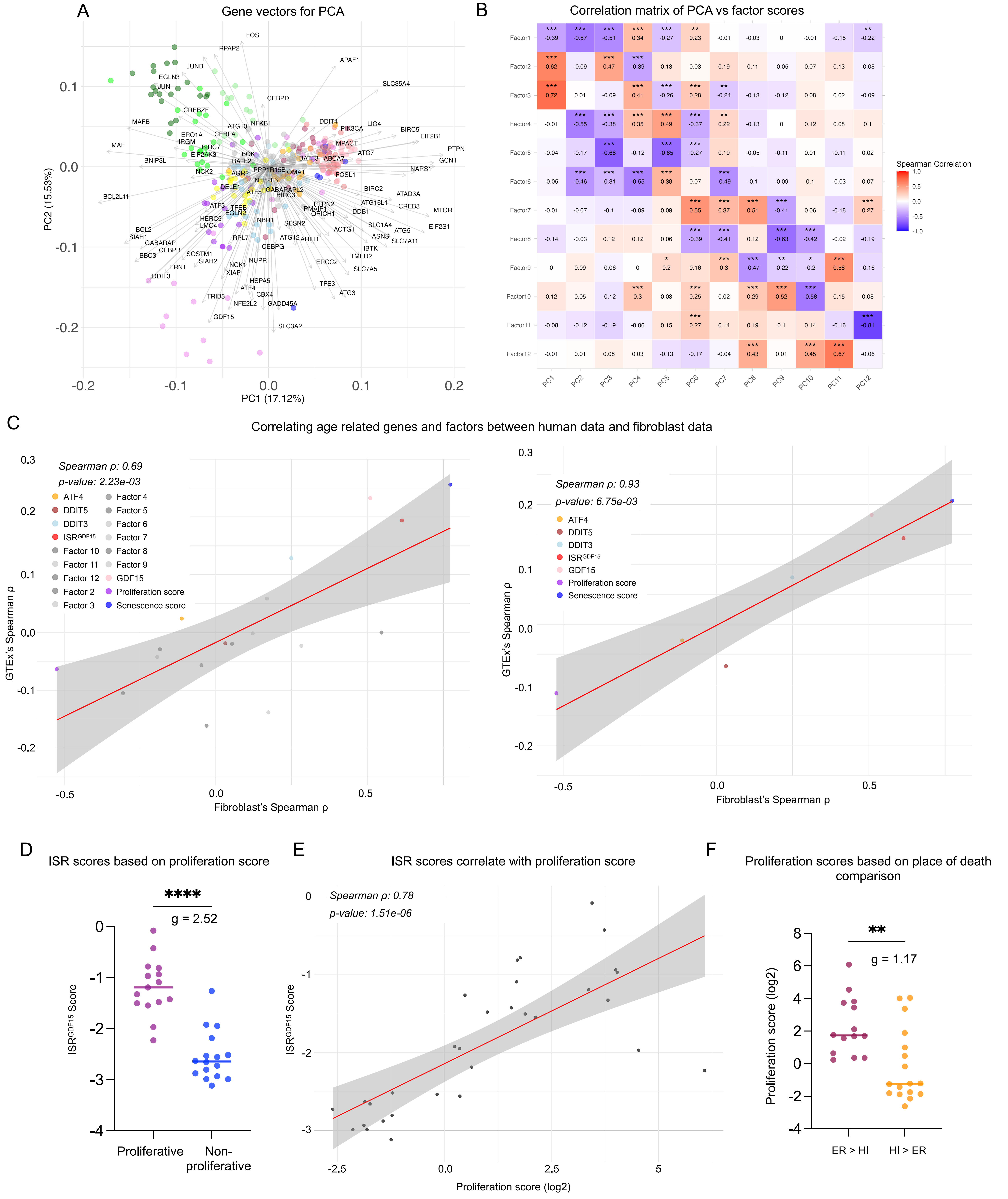

Supplement: Supplementary file 1 [file media-1.pdf]
